# Diversity, Lifestyle, Genomics, and Their Functional Role of *Cochliobolus*, *Bipolaris*, and *Curvularia* Species in Environmental Remediation and Plant Growth Promotion under Biotic and Abiotic Stressors

**DOI:** 10.3390/jof9020254

**Published:** 2023-02-14

**Authors:** Nasir Ali Khan, Sajjad Asaf, Waqar Ahmad, Rahmatullah Jan, Saqib Bilal, Ibrahim Khan, Abdul Latif Khan, Kyung-Min Kim, Ahmed Al-Harrasi

**Affiliations:** 1 Natural and Medical Science Research Center, University of Nizwa, Nizwa 616, Oman; 2 Department of Plant and Soil Science, Institute of Genomics for Crop Abiotic Stress Tolerance, Texas Tech University, Lubbock, TX 79409, USA; 3 Department of Engineering Technology, University of Houston, Sugar Land, TX 77479, USA; 4 Department of Applied Biosciences, Kyungpook National University, Daegu 41566, Republic of Korea

**Keywords:** *Curvularia*, *Bipolaris*, fungi, phytohormones, abiotic stress, biocontrol, enzymes, bioremediation, diverse role

## Abstract

*Cochliobolus*, *Bipolaris*, and *Curvularia* genera contain various devastating plant pathogens that cause severe crop losses worldwide. The species belonging to these genera also perform a variety of diverse functions, including the remediation of environmental contaminations, beneficial phytohormone production, and maintaining their lifestyle as epiphytes, endophytes, and saprophytes. Recent research has revealed that despite their pathogenic nature, these fungi also play an intriguing role in agriculture. They act as phosphate solubilizers and produce phytohormones, such as indole acetic acid (IAA) and gibberellic acid (GAs), to accelerate the growth of various plants. Some species have also been reported to play a significant role in plant growth promotion during abiotic stresses, such as salinity stress, drought stress, heat stress, and heavy metal stress, as well as act as a biocontrol agent and a potential mycoherbicide. Similarly, these species have been reported in numerous industrial applications to produce different types of secondary metabolites and biotechnological products and possess a variety of biological properties, such as antibacterial, antileishmanial, cytotoxic, phytotoxic, and antioxidant activities. Additionally, some of the species have been utilized in the production of numerous valuable industrial enzymes and biotransformation, which has an impact on the growth of crops all over the world. However, the current literature is dispersed, and some of the key areas, such as taxonomy, phylogeny, genome sequencing, phytohormonal analysis, and diversity, are still being neglected in terms of the elucidation of its mechanisms, plant growth promotion, stress tolerance, and bioremediation. In this review, we highlighted the potential role, function, and diversity of *Cochliobolus*, *Curvularia*, and *Bipolaris* for improved utilization during environmental biotechnology.

## 1. Introduction

Fungi are eukaryotic organisms that inhabit living plants and animals or dead tissues. They are important sources of food, alcohol, enzymes, antibiotics, organic acids, and plant growth-promotor compounds. Fungi are primary decomposers of substances in the environment and are distinctive from other living organisms. They magnificently decompose organic waste material and very quickly target cellulose, lignin, gums, and other organic complex substances. Fungi are essential for a number of physiological activities, including mineral and water uptake, chemical changes, stomatal movement, and the biosynthesis of auxins, lignans, and ethylene, which assist plants in overcoming environmental stresses, such as drought, salt, heat, cold, and heavy metals [1].

Endophytic fungi residing inside aerial plant tissues at least once during their lifetime do not produce any apparent harm to the host plant [2]. Fungal endophytes promote plant growth in a variety of ways, including the release of plant growth hormones, such as cytokines, indole acetic acid, or gibberellins, and by providing biologically fixed nitrogen [3,4,5,6,7,8]. The host plants can benefit from these plant hormones or secondary metabolites during biotic and abiotic stressors [9]. The interrelating partners are unaffected in this interaction, and the individual advantage is dependent on both interacting partners [10]. Even in stressful situations, plant growth-promoting endophytic fungi are beneficial to host plants [11]. These fungi influence the main features of plant physiology and host defense against various biotic and abiotic stresses [11]. Fungal endophytes have been studied for their function to improve plant promotion and also play a role in several processes, such as nitrogen fixation and phosphate solubilization [6].

Fungal biological control is a fascinating and fast-evolving field of study with significance for plant productivity. To attract nutrients from the environment to colonize plant tissues and thrive in the plant–microbe connection, endophytic fungi must produce enzymes. Particularly, a soil fungus produces a number of fungal enzymes that have significance in the food, biofuel, paper, detergent, animal feed, textile, leather, and pharmaceutical industries [12]. The usage of fungi to eliminate environmental pollutants has attained advancement in recent years, but most studies have focused on white rot fungi and endophytic fungi [13,14]. The use of endophytic fungi in the remediation of harmful pollutants, such as hydrocarbons, polychlorinated biphenyls (PCBs), polyaromatic hydrocarbons (PAHs), radionuclides, and metals, could be a significant way and novel approach.

## 2. Diversity, Habitation, and Ecology of *Cochliobolus*, *Curvularia*, and *Bipolaris*

The *Cochliobolus*, *Bipolaris*, and *Curvularia* species are found all over the world as pathogens and saprobes in plants, humans, and animals [15,16]. *Curvularia* species have also been found in the air [17], in aqueous habitats and in soil [16,18]. These species were reported primarily based on Poaceae members and are major pathogens of grass and staple crops, such as rice, maize, wheat, and sorghum [19]. *Caricaceae*, *Actinidiaceae*, *Convolvulaceae*, *Aizoaceae*, *Iridaceae*, *Polygonaceae*, *Lamiaceae*, *Oleaceae*, *Lythraceae*, *Fabaceae,* and *Rubiaceae* are among the other genera that serve as hosts [15,20]. Most *Curvularia* species, on the other hand, are found as facultative plant pathogens in tropical and subtropical environments, as well as endophytes on several Sudanese plants [21,22]. *Curvularia* also includes new human opportunistic pathogens, such as *C. chlamydospora* and *C. lunata,* which cause infections of the respiratory tract, cutaneous, brain, and ocular surfaces, primarily in immunocompromised people [23]. Other species, such as *C. hawaiiensis*, *C. australiensis*, *C. spicifera*, and *C. lunata*, have also been isolated from human specimens and are thought to be the cause of animal and human disorders [24]. Two more species, *C. hominis* and *C. tuberculata*, have recently been identified as causes of keratitis [25] and a human disseminated phaeohyphomycosis [26]. *Curvularia* spp. has replaced several species previously classified as *Pseudocochliobolus*. As a result, *Pseudocochliobolus* is not further considered a distinct genus, as the type has been synonymized with *Curvularia* [16]. In this review, we discuss the diversity of *Cochliobolus*, *Bipolaris*, and *Curvularia* in different environments, the potential role of these genera in biocontrol, biotransformation, and beneficial metabolite production. We will discuss how this species play role in bioremediation, phosphate solubilization, and abiotic stress tolerance in plants.

*Cochliobolus* species are heterothallic fungi that thrive in soil and organic matter and as endophytes, producing secondary metabolites with important biological functions [27,28]. *Curvularia* [29] is a hyphomycete fungus that is both cosmopolitan and widespread. *Curvularia* is distinguished by the development of brown distoseptate conidia, which have paler terminal cells and inordinately larger intermediate cells, contributing to the curvature of the organism. It is worth noting that fungal endophytes are responsible for more than half of all bioactive substances discovered [30]. Many medicinal chemicals are derived from endophytic fungi, including those with anticancer, antifungal, antiviral, antibacterial, antitumor, and anti-inflammatory properties. *Cochliobolus* is a fast-growing fungus that may reach a diameter of 5.5 cm in just five days when plated on malt extract and Czapek’s Agar media [31,32]. These species are weed pathogens, and because weeds and pathogens have evolved together over time, they can be used as weed herbicides [19].

## 3. Classification, Nomenclature, and Phylogenetic Analysis

**Figure 1:**
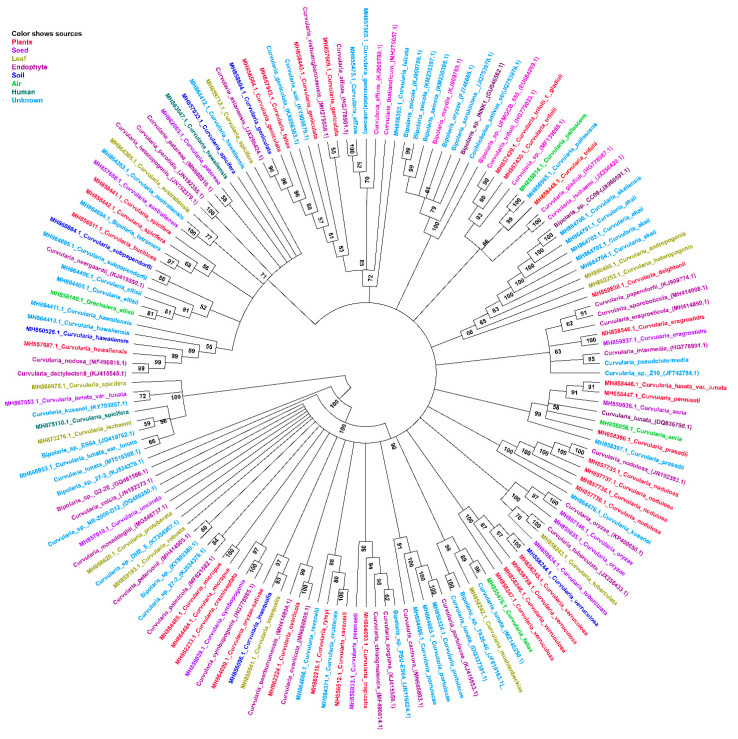
Phylogenetic analysis of the *Curvularia* and *Bipolaris* species isolated from different sources based on ITS genes. The maximum likelihood (ML) method was used to infer the phylogenetic analysis. The number above the branches is the bootstrap value for the ML.

The *Cochliobolus*, *Curvularia*, and *Bipolaris* genera belong to the phylum Ascomycota, class Dothideomycetes, order Pleosporales, and family Pleosporaceae. The taxonomy of *Cochliobolus* has become rather confusing as a result of the numerous nomenclatural changes that have occurred in the sexual and asexual stages of species during the past few decades. For mycologists and plant pathologists, frequent name changes and taxonomic revisions have produced some confusion [15]. Because many *Curvularia* species share similar features and have overlapping conidial dimensions, identifying species merely based on morphology is challenging. Accurate and updated taxonomy of this genus and precise species identification are essential for disease control, plant breeding, and the implementation of National Plant Protection Organization (NPPO) measures. [33]. There are now 238 *Curvularia* species (excluding duplicated), 66 *Cochliobolus* species, and 142 *Bipolaris* species available in the MycoBank database (Table S1). Previously there were 81 recognized species for which DNA barcodes have been used to establish taxonomic placement, allowing for reliable identification and comparison [16]. The evolutionary relationships of *Cochliobolus*, *Curvularia*, and *Bipolaris* [34] have determined phylogenetic analyses using rDNA internal transcribed spacer (ITS) regions and glyceraldehyde 3-phosphate dehydrogenase (GAPDH), large subunit (LSU), and translation elongation factor 1-α (TEF1) gene sequences. Additionally, the use of molecular phylogenetic analysis based on multiple loci as a tool to identify novel species has increased [35,36]. The asexual genera *Bipolaris* and *Curvularia* lack a distinct morphological borderline, and some species exhibit intermediate morphology. Here, we retrieved the ITS sequences of those species from the NCBI database that may be involved in promoting plant growth, producing metabolites, biological control, symbiotic associations, and biotransformation. The substrate and source (host) information was retrieved from the Westerdijk institute of the Fungal Biodiversity Culture center (https://wi.knaw.nl, accessed on 15 April 2022). All the sequences were aligned to each other using ClustalX v. 1.83 [37]. Phylogenetic analyses were performed using MEGA X [38], and the maximum likelihood (ML) method based on the Tamura-Nei model, as reported previously [39], was used to construct the phylogenetic tree. The robustness of branches was assessed through the use of bootstrap analyses of 1000 replicates. The findings revealed that several *Bipolaris* species formed clades with *Curvularia* species (Figure 1, Table S2). The species isolated from different source are clustered together. The endophytic *Curvularia* species are clustered with other species relevant to biological control. However, there is no obvious difference between *Curvularia* and *Bipolaris* species in terms of their functioning based on the ITS region.

## 4. Cultivation and Preservation in Culture

Pure culture methodology enables researchers to find, isolate, identify, and quantify the amount and types of fungus found in various habitats and the nutritional, chemical, and environmental needs for their growth and metabolism. Gene sequence data are also frequently utilized to solve taxonomic difficulties at the genus and species levels [40]. Sometimes such studies are defective since they do not include ex-type cultures of the species or genus being studied [41]. If ex-holotype, ex-isotype, or ex-epitype cultures exist, they must be sequenced so that species names can be applied consistently [42]. Here, Tables S2 and S3 show lists of *Chochliobolus*, *Curvularia*, and *Bipolaris* species found in the Westerdijk institute of the Fungal Biodiversity (https://wi.knaw.nl/page/About, accessed on 15 April 2022), as well as in the MycoBank databases (https://www.mycobank.org, accessed on 15 April 2022). Sometimes, when type culture or type-derived sequences were not available, the culture sequences received from the original author or authorized with efficient methods were used instead. In future studies, the type of derived sequences also shown in Table S3 can be employed to examine and identify new specimens in this genus.

## 5. The Pleomorphic Genus *Cochliobolus*

Pleomorphic fungi are allowed to have two names under the International Code of Botanical Nomenclature. Nonetheless, a worldwide goal is to give all fungi a single name [43]. Many *Cochliobolus* species have asexual stages in either *Bipolaris* or *Curvularia* and thus are synonyms. *Cochliobolus heterostrophus* and *B. maydis* (Y. Nisik. & C. Miyake) Shoemaker, for example, are biologically the same species, and *Cochliobolus geniculatus* R. R. Nelson and *Curvularia geniculata* (Tracy & Earle) Boedijn are biologically the same species. *C. boutelouae, C. miakei*, *C. palmivora*, *C. sasae*, and *C. sitharamii* are the only species not connected to any anamorph state [44]. A *Cochliobolus* teleomorph was reported in *B. micropus* (Drechsler) Shoemaker, but it was never named or characterized, and its current name is *C. micropus* in the Index Fungorum database. According to an updated list in the Index Fungorum database there are 140 *Bipolaris* names, 54 *Cochliobolus* names, and 233 *Curvularia* names. Only 43 *Cochliobolus* teleomorphs are connected to the asexual states of *Bipolaris* (11 names) and *Curvularia* (18 names) [45]. The authors of [34] investigated nine *Curvularia* and *Bipolaris* species with unknown sexual states and discovered that they all share an ancestor with the sexual *Cochliobolus* species. Molecular analysis of *Bipolaris* and *Curvularia* species revealed that none of them were monophyletic [34]. When picking a unique name for a fungus species, there are several points of view based on which the name should be used, such as the oldest name, the teleomorphic name, or the most important name [46]. The name *Curvularia* [29] is older than *Bipolaris* [47] and *Cochliobolus* [48]; it may be necessary to use *Curvularia* for all species of these genera after the Botanical Code is updated in 2013 and Article 59 is no longer in effect.

## 6. Morphology of *Curvularia* and *Bipolaris*

**Figure 2:**
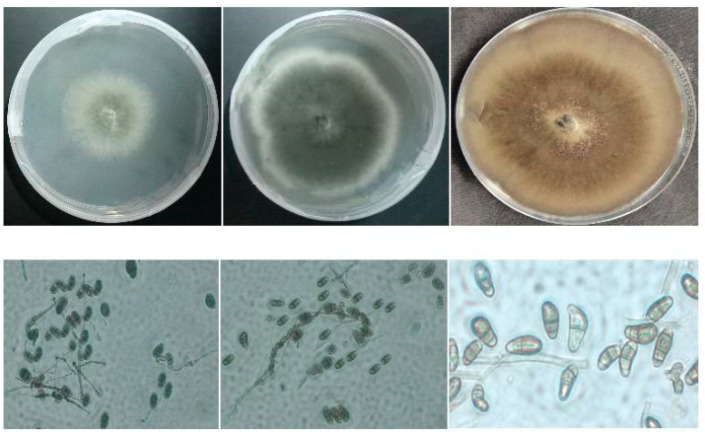
Morphological observations of *Curvularia* and *Bipolaris* species on agar plates (Petri dish), and microscopic observations on wet preparation glasses with a zoom of 20 × 10 using a computer microscope.

*Cochliobolus* ascomata have a globose body and are dark brown to black in color. On the ascomata, hyaline-to-brown sterile hyphae and conidiophores are common [15]. Characteristics include bitunicate asci, 2–8-spored, cylindrical-to-obclavate or obclavate cylindrical. The ascus contains filiform ascospores that are more or less coiled in a helix. Most *Cochliobolus* species produce sterile protothecia (sclerotia) that lack ascogenous hyphae [47] (Figure 2). It is a saprophyte that lives mostly in the form of thick-walled conidia. It can also survive as a mycelium in soil or crop debris. In the disease cycle, the sexual stage is less important. Mycelium from infected seeds, conidia in the soil, and conidia on the kernel surface are all examples of the primary inoculum [45]. Conidia of diverse forms were found in *Cochliobolus* species, such as straight conidia, curved conidia, smooth conidia wall, curved conidia with 3-distoseptate, a tuberculate conidia wall, conidia with 5-distoseptate, and conidia with 6- to 10-distoseptate. The wall is made up of cells with the same or less body density. Asci are two to eight-spored, bitunicate, tubular to obclavate, or obclavate tubular [15]. In the ascus, ascospores are filamentous and spiral in a helix [15]. When the generic reports of *Bipolaris* and *Curvularia* are compared, the two taxa are morphologically very similar and cannot be distinguished using any taxonomic approach [15]. Furthermore, there are physical differences between these two taxa, such as septal structure; *Curvularia* species have euseptate conidia, whilst *Bipolaris* species have distoseptate conidia [49].

## 7. *Cochliobolus*, *Curvularia* and *Bipolaris* Lifestyle

### 7.1. Cochliobolus, Curvularia and Bipolari as Epiphytes

Epiphytic microorganisms live on the surfaces of aerial plant components, and many of them can affect pathogen growth [50]. Epiphyte–endophyte interactions have crucial implications for fungal biodiversity and plant health [51]. *Curvularia* and *Bipolaris* species have only been found as epiphytes in plants in a few cases. For example, *Lasiodiplodia theobromae*, a common fungal disease, is antagonized in vitro by *C. pallescens*, which was isolated as an epiphytic from the surface of banana fruit [52].

### 7.2. Cochliobolus, Curvularia, and Bipolari as Endophytes

As shown in Table 1, numerous *Cochliobolus* species have been found to be endophytes of diverse plant species, and their percentage frequency as compared to that of other species is often modest [53,54]. In wheat glumes, *B. sorokiniana* (sexual state: *C. sativus*) was found in high numbers [55]. Endophytes are commonly seen in the asexual stages of *Bipolaris* and *Curvularia*; however, there are rare reports of the teleomorph phase as endophytes. Recently, *C. lunata* strain AR11 was identified as an endophyte that was employed to improve rice plant growth and reduce salt and drought stress [56]. Furthermore, *C. iranica* a new endophytic species, was also isolated in Iran from ornamental trees [57]. Several *Curvularia* endophytes are latent pathogens in plants under stress, and they cause severe illness in Musa spp. [58]. The transformation of the life mode from endophyte to pathogen might be triggered by changes in host plant sensitivity, high humidity, or a lack of nutrients [59]. *C. protuberata* was also reported as an endophyte of *Dichantelium lanuginosum*, a form of a three-way symbiotic interaction with host plants, allowing them to live under harsh soil conditions [60]. Asexual stages of *Bipolaris* are common in marine sponges and can also be seen in combination with sea grasses [61]. The coastal sponge *Gelliodes carnosa* was found to be connected with a *Bipolaris* species that had close affinity with the widely-known plant pathogen *B. sorokiniana* [62].

### 7.3. Cochliobolus, Curvularia, and Bipolaris as Saprobes

As shown in Table 2, *Cochliobolus* species and their asexual forms have also been isolated as saprobes [81,82]. *Cochliobolus* anamorphic states have been identified in conjunction with a variety of Poaceae species, including bamboo and other host plants. Various researchers have reported about 19 *Cochliobolus* species and their asexual forms from different plant dead materials (Table 2). *C. lunata*, for example, is often seen as a bamboo clump saprobe [83]. Various *Cochliobolus* species and their asexual forms have been isolated from several dead wood plants [83,84].

## 8. Application of *Cochliobolus*, *Curvularia,* and *Bipolaris* in Agriculture and Functional Roles

### 8.1. As a Plant Growth Promotor

Several species of the *Cochliobolus* genus have been shown to promote plant growth. Compounds synthesized by *C. setariae* IF0 6635 promoted rice seedling elongation (Table 3). The active compound designated as cis-sativenediol was isolated from both fungal mycelia and culture filtrates with several related metabolites, including its trans isomer. It is interesting that pathogenic fungi containing growth-inhibitory substances for host plants can also produce a plant growth-promoting substance. A fungal elicitor extract was used to test the growth-promoting effects of *C. lunatus* and *C. pallescens* recovered from the *Artemisia annua* L. plant [93]. Some chemical compounds produced by or found in endophytic fungus may be responsible for the growth-promoting effects of the fungal elicitor extract [94]. *Arabidopsis thaliana* (L.) Heynh. seedlings grew better when given a cell wall extract from an endophytic fungus [95]. As reported in other fungal species, there is a high concentration of chitin and chitosan in the cell walls of *Cochliobolus* fungi [96]. Artemisinin production in *A. annua* plants was activated by chitosan applied topically (foliar application) [97]. *Abelmoschus esculentus* (L.) Moench, grew taller, had more leaves, and produced more fruit due to this treatment [98]. Fungal elicitor extract, in addition, has the potential to induce stress responses in plants, making them more resistant to infections and other environmental challenges. Some *Cochliobolus* species have been shown to promote plant development by producing phytohormones, through phosphate solubilization, and other metabolite production, as discussed below in detail and as shown in Table 3.

### 8.2. Phytohormone Production

Phytohormones are chemical messengers that influence the development of plants. At low concentrations, these compounds can control the metabolic activity of plants and have a wide variety of uses in agriculture [105]. One of the most important phytohormones, indole acetic acid (IAA), controls cell growth and division [106]. The plant-derived IAA is also produced by soil and endophytic microorganisms and has been shown to enhance plant development [107,108]. Endophytic fungi, such as *Curvularia* species, have recently been found to produce IAA in the tissues of aerial plant parts [109]. These fungi produce IAA, which has been demonstrated to increase plant biomass and root growth. The ability of *C. geniculate* to produce IAA was discovered in several experiments by enhancing plant growth [2,56,110,111]. When exposed to salt and drought stress, the *C. lunata* AR11 strain has the capacity to spontaneously synthesize the GAs (GA_1_, GA_3_, GA_4_, and GA_7_) and organic acids needed to increase nutrient absorption [56,112]. Similarly, *Bipolaris* sp. CSL-1 was reported to promote plant growth by producing IAA and GAs [113].

### 8.3. Phosphate-Solubilizing Agent

All plant parts contain endophytic fungi, and the solubilization of soil nutrients and the synthesis of phytohormones are numerous ways these fungi help plants grow [114]. Under harsh environmental circumstances, these fungi promote plant growth and health [110]. The hydrolytic enzymes produced by these fungi in the breakdown of organic matter have been studied in a few studies, but there are no studies on their involvement in the solubilization of insoluble nutrients in the soil and the availability of those nutrients to plants [115]. An essential macronutrient for plant development and growth is phosphorus (P). Tropical soils, on the other hand, have low P availability [116]. These complexes are insoluble because the negatively charged inorganic phosphate anion rapidly interacts with cations, such as iron, calcium, and aluminum. As a result, in alkaline soils, phosphate is often found as the tricalcium phosphate [Ca_3_(PO_4_)_2_], whereas in acidic soils, it is found as FePO_4_ and AlPO_4_ [117]. Plants cannot readily access certain types of phosphate. As a result, synthetic P fertilizers are required to maximize crop productivity on soils like these. It is also worth noting that synthetic fertilizers have several negative impacts on ecosystems. In order to limit the usage of synthetic fertilizers, one alternate technique is to use soil microorganisms, particularly those capable of solubilizing insoluble nutrients. Research has shown that fungi are more effective than other soil microorganisms in breaking down nutrient bonds [118]. Researchers found that fungal isolates solubilized Ca_3_(PO_4_)_2_ and rock phosphate more effectively than bacteria [119]. Phosphate rock may be more easily dissolved by the dark septate endophyte fungus [100]. Phosphate solubilization by *Curvularia* and *Bipolaris* strains has been found to promote plant growth [56,120]. Mixing non-antagonistic *Bipolaris* species in a bio-fertilizer formulation might be essential in improving Al–PO_4_-H_2_O solubilization and boosting soil fertility. The fungus showed promising characteristics for use as biofertilizers in agricultural acreage sustainability management. The root endophytic fungus *C. geniculata* from *Parthenium* Hysterophorus roots enhanced plant development via phosphate solubilization and solubilized FePO_4_ and AlPO_4_ more effectively than the easily solubilized Ca_3_(PO_4_)_2_ phosphates [101].

## 9. *Bipolaris* and *Curvularia* Secondary Metabolite Production

*Cochliobolus* and its anamorph generate a wide range of secondary metabolites. Toxins and metabolites produced by taxa cannot be used to distinguish between both the species *Bipolaris* and *Curvularia* [15]. Curvulin, for example, is derived from *B. papendorfii* and *Curvularia* sp., among other sources [15]. Some examples of novel metabolites and toxins from *Cochliobolus* strains are listed in Table 4. Phytotoxic, cytotoxic, leishmanicidal, antioxidant, fungicidal, and antibacterial properties were found in many crude extracts of *Bipolaris* and *Curvularia* (Table 4). It is possible that crude extracts of *Bipolaris* and *Curvularia* spp. might be used to treat various chronic disorders and might be beneficial for agriculture and pharmaceuticals. These secondary metabolites (SMs) come from various chemical families, including peptides, terpenes, quinones, alkaloids, polyketides, and anthraquinones, as shown in Table 4. *Cochliobolus carbonum* produces EXG1p novel Exo-1,3-glucanase, a cell wall-degrading enzyme [121]. A Gamma pyrone [122] plant growth inhibitor and 6-Chlorodehydrocurvularin are produced by *Cochliobolus spicifer* [123]. These fungi synthesize IAA, which has been demonstrated to increase root growth and plant biomass [112]. The ability of *C. geniculate* to produce IAA was discovered in several experiments by enhancing plant growth [101]. When exposed to salt and drought stress, the *C. lunata* AR11 strain has the capacity to spontaneously synthesize the GAs (GA_7_, GA_4_, GA_3_, and GA_1_) and organic acids needed to increase nutrient absorption [56]. Cochlioquinones A and B, novel metabolites having a p-quinonoid origin, are generated by *Cochliobolus miyabeanus* [124]. The 9-hydroxyprehelminthosporol chemical generated by *Cochliobolus sativus* is an antiviral. Additionally, *Cochliobolus* exhibits other actions that may be beneficial to plants, including being a widespread disease of grasses (Poaceae) and essential food crops, such as rice, wheat, and maize [45]. *Cochliobolus*, for example, may be utilized as a biochemical modulator to relieve okra plant salinity stress [70,99]. Buffelgrass is poisonous to chloromonilinic acids C and D from *Cochliobolus australiensis*, which delay germination and drastically inhibit radicle development [125].

## 10. Biological Activities of the Genera *Curvularia* and *Bipolaris*

*Cochliobolus* species have also shown promise in a variety of biological functions. The metabolite extract or secondary metabolites, e.g., natural products from the genera *Curvularia* and *Bipolaris*, have powerful biological activity, such as antimicrobial (antibacterial and antifungal), antioxidant, leishmanicidal, cytotoxic, and phytotoxic effects, as shown in Table 5. *Cochliobolus* metabolites were found to have antileishmanial properties against *Leishmania* and *Trypanosoma*. *Leishmania amazonensis* amastigote-like forms were killed by the crude extract of *Cochliobolus* sp. at 20 g/m concentration, and this action might be linked to the metabolites cochlioquinone A and isocochlioquinone A [70,154] (Table 4). *Cochliobolus* metabolites were shown to have anticancer effects as well. Radicinin extracted from *Cochliobolus geniculatus* WR12 has an IC50 of 25.01 ppm against T47D cells [155]. Dendryphiellin I, which was isolated from the marine *C*. *lunatus* SCSIO41401, was discovered to be cytotoxic to three renal cancer cell lines (ACHN, 786-O, and OS-RC-2), a human liver cancer cell line (HepG-2), and a human gastric cancer cell line (SGC7901). We observed antibacterial activity for dendryphiellin I in marine bacteria *Curvularia lunata* against three distinct bacteria (MIC 1.5–13 g/mL).

## 11. Role of *Cochliobolus*, *Curvularia,* and *Bipolaris* Species in Abiotic Stress Tolerance

### 11.1. Salinity Stress Tolerance

*Cochliobolus* exhibits different activities that can be useful for plants. For example, *Cochliobolus* can be used as a biochemical modulator to alleviate salinity stress in okra plants (Table S4) [70,99]. In many parts of the world, salinity is one of the major abiotic stressors that restricts plant growth, development, and improvement and causes an excessive decline in plant productivity [7]. Moreover, a quarter of the world’s irrigated land suffers from salinity stress, making it one of the most critical environmental issues. Three different physiological stressors are applied to plants grown in salty environments. The earliest harmful effects of the sodium and chloride ions, which are present in salty soils, interfere with the structure of enzymes and other macromolecules, impede respiration and photosynthesis, damage cell organelles, cause iron deficiency, and inhibit protein synthesis [164]. Plants in salty soils are more susceptible to physiological dehydration because they must limit water transfer from roots to soil to maintain a lower internal osmotic potentials [165]. Finally, salty soil inhibits nutrient absorption in plants, resulting in an imbalance in plant nutrition. This kind of stress causes cellular death-causing reactive oxygen species (ROS) in plants. At such times, free electrons from electron transport chains in chloroplasts and mitochondria are provided by reactive oxygen species (superoxide, singlet oxygen, and hydrogen peroxide) and hydroxyl radical [166]. *C. lunatus* boosted okra plant growth and biomass while reducing lipid peroxidation in NaCl-treated plants. *C. lunatus* enhanced the amounts of flavonoids, phenolics, phytohormones, total chlorophyll, and proline in okra plants treated with NaCl [70,99].

### 11.2. Enhance Heat and Drought Stress Tolerance

As a result of the global warming caused by greenhouse gas emissions, which are expected to rise by 1.5 to 8.8 degrees Celsius by the year 2100 [167], there is a serious threat to food and nutritional security worldwide. Plants in their ecological niches must either evolve natural mechanisms or be genetically engineered with heat shock genes and cognates or specific non-protein-coding short RNAs to withstand heat stress and drought. When plants are exposed to both positive and negative stimuli, their genomes can sense them and regulate adaptability to selection forces with great care to ensure survival in a changing biota [168]. Soil temperature resistance may be improved by fungal symbiosis, as indicated in Table S4, by *C. cryptic* [169]. Tolerant of constant temperatures of 50 degrees Celsius and periodic soil temperatures of 65 degrees Celsius, *Dichanthelium lanuginosum* from geothermal soils in US national parks can survive for ten days due to a mutualistic relationship with *C. protuberata* [169]. When looking into geothermal habitats, scientists identified *C. crepinii* and *C. protuberrata*, both of which are attracted to the plant *Hedyotis diffusa*, and successfully boosted the thermostability [170]. Similarly, under controlled laboratory conditions, all *C. crepinii* and *C. protuberrata* isolates grew at 50 °C and gave 5 days of thermotolerance to *Oryza sativa* [170]. In spite of [169] showing a complicated link between *Curvularia* thermal tolerance virus (CThTV) and *C. protuberrata*–*D. lanuginosum* thermotolerance [169,170] various degrees of thermotolerance to their different hosts were found in *Curvularia* species in their biological habitat. Melanin of most *Bipolaris* and *Curvularia* species is expected to play a significant role in distributing heat along hyphae and sequestering oxygen radicals produced during heat stress [45,171,172]. Osmoprotectants, such trehalose, glycine betaine, and taurine production, have been related to virus-induced heat tolerance in *Curvularia* [60]. Fungi that produce melanin are also tolerant to abiotic stresses, such as high temperature, chemicals, radioactive pollution, solar radiation, and drought [173]. Recent research found that co-culturing pre-germinated rice seedlings with a thermotolerant endophyte improved root and shoot development under high-temperature stress [100]. Pre-germinated rice seedlings were infected with an endophytic thermotolerant fungus, which increased root length throughout the seedling phase [174]. *C. lunata* AR11 strain was recently identified as a plant growth-promoting fungus by producing phytohormones and alleviating drought and salinity stress in plants [56].

## 12. *Curvularia* and *Bipolaris* Biotechnological Applications

Many countries are doing their research to explore the beneficial potential of *Cochliobolus*, *Curvularia*, and *Bipolaris*. *Cochliobolus* species that are endophytic may be employed to reduce weeds, plant diseases, and insect pests [175,176]. Many *Cochliobolus* and *Bipolaris* species are also used in biotransformation and antibiotic synthesis and have a wide range of biological activity.

### 12.1. As a Biocontrol Agent

The relevance of an endophytic fungus as a plant biocontrol agent has been proven in several studies. Fungi as biological control agents are a rapidly growing area with implications for food security, human health, and animal and plant productivity [177]. Weeds are a cost-cutting measure in agricultural production [178]. Plant pathogens are now widely accepted as a viable, safe, and ecologically beneficial weed-management technique for agroecosystems. Mycoherbicides are important in the transition to organic farming and in reducing the usage of chemical herbicides. Table 6 shows some evidence that *Curvularia* and *Bipolaris* might be used as potential mycoherbicides. Many *Cochliobolus* species cause weed diseases that have shown to be effective as weed herbicides and have been found in several tests to be effective mycoherbicides [19]. Infections may have developed biochemical methods to destroy the weed host, and *Bipolaris* sp. was tested in Australia as a potential herbicide against serrated tussock [179]. *Cochliobolus* strains have been found to contain curvularides, cochlioquinones, anthroquinones, and several new proteins associated with cyclic peptide control and cell wall disintegration. These chemicals could have significant pharmacological qualities, such as antifungal capabilities, and hence could be applied in medical research [131]. Invasive weed control is a serious difficulty in a mono-cropping system, since it reduces crop production and quality, causes allergic reactions after pollination, and causes aesthetic discomfort [180,181].

With the increased use of endophytic fungi proficiently producing phytotoxic poisons and thus gradually replacing synthetic herbicides, a paradigm shift has been noticed [27]. *Curvularia* species are an essential source of mycoherbicides because they produce a wide range of bioactive compounds [176]. *C. eragrostidis*, for example, produces phytotoxic chemicals, such as dehydrocurvularin, helminthosporin, and curvularin, which are used to suppress a variety of weeds [185]. While *C. eragrostidis* triggers plant diseases [189], it was discovered that dehydrocurvularin inhibits reoxidation of the photosynthetic chain’s primary electron acceptor (QA) [190]. Helminthosporin, on the other hand, attacks the chloroplast function of the common weed *Digitaria sanguinalis* [190].

### 12.2. Cochliobolus, Curvularia, and Bipolaris Species in Enzyme Production and Biotransformation

A filamentous fungus mostly produces commercial and industrial enzymes. In 2018, industrial enzymes were expected to be valued at USD $5.6 billion [100]. In 2017, industrial enzymes had a 26 percent (USD $1.4 billion) impact on food and drinks, 18 percent (USD $969.3 million) in biofuels, and 14 percent (USD $754.4 million) in detergents. The demand for additional industrial enzymes is predicted to reach USD $7.7 billion by 2024 as a consequence of changing lifestyles and rising waste generation throughout the globe [100]. In contrast to other fungi, *Bipolaris* and *Curvularia* species synthesize extracellular enzymes radially, making them optimal for enzyme synthesis [191]. When compared to proteins in protein databases, almost 25.64 percent of *Cochliobolus lunatus* proteins have no putative activities, according to a secretomics analysis [100]. According to quantitative evidence, *C. lunatus* produces laccase and manganese (Mn) peroxidase at the same time [192]. According to a genome-wide study, single-copy gene loss in certain *Pleosporales* species allows them to produce manganese (Mn) peroxidase, which is generally conserved in *Basidomycetes* [193]. In *Pleosporale* order species (*Pyrenophora tritici-repentis*, *Stagonospora Nodorum*, and *C. heterostrophus*), only a single copy of the CCC1 gene, a Mn^2+^ and Fe^2+^ vacuolar transporter, was discovered [193]. However, it has been proposed that measuring laccase production might be as simple as combining suitable *Bipolaris* species or strains in the same microenvironment. *Curvularia* and *Bipolaris* species have been shown to generate bioactive antimicrobial chemicals that are used in enzyme manufacturing and biotransformation, as shown in Table S5.

## 13. In Bioremediation and Waste Biomass Valorization

In both rich and emerging economies, poor industrialization, agricultural, and environmental policies have resulted in massive discharges of toxic waste, such as fluorinated and chlorinated aromatic hydrocarbons. Furthermore, laccase-producing organisms, such as genetically modified plants that overexpress laccase and free-living *Curvularia* and *Bipolaris* species, are used instead of costly physical and chemical detoxification methods [194,195]. Bringing these “nature bioengineering” fungus species on board and maximizing their capabilities could have ramifications in medicine, agriculture, and the chemical sector, ensuring the bio economy’s long-term maintenance and viability.

It is important to fully comprehend the economic benefits of the cryptic *Cochliobolus* complex at the organismal level, such as their role in the production of industrial enzymes [195], medications [196], and bioremediation [194], biodiesel [197]. Bioremediation is the capacity of microorganisms to break down (detoxify) harmful chemicals and undesirable organic compounds into non-toxic molecules [198]. In the agro-industry, palm oil extraction creates much bio waste, such as empty fruit bunches and palm oil mill effluent (POME). Palm trash is often disposed of by burning, which pollutes the environment. A mixture of ligninolytic enzymes (manganese peroxidase, lignin peroxidase, and laccase) and glycosylhydrolytic enzymes (xylanase and cellulase) is produced during POME–*Curvularia clavata* interactions [199]. In the instance of bioremediation, *C. lunatus* was found to biodegrade crude oil, resulting in a 1.5 percent weight loss (of the crude) in one week, 2.1 percent weight loss in two weeks, and 4.7 percent weight loss in three weeks [100,200]. Similarly, the fungus *C. lunatus* strain CHR4D was isolated from crude oil-polluted shorelines in India, and it was determined that chrysene (C_18_H_12_), a four-ringed high molecular weight polycyclic aromatic hydrocarbon, was degraded swiftly and effectively [201]. Surprisingly, after only four days, 93.10 percent of the chrysene was eliminated [201]. Unlike the use of *C. lunatus* to break down plastic [194], co-substrate, such as glucose and tartrate, were used to promote the production of extracellular lignin-modifying enzymes (LMEs) during chrysene metabolism, enhancing the fungus’ efficiency [201]. LMEs that may co-metabolize ringed high molecular weight polycyclic aromatic hydrocarbons to speed up decomposition include peroxidases, manganese peroxidases, and laccases [202].

Cochliotoxi isolated from *C. australiensis* has been proven to act as a bioherbicide [27]. *Curvularia* species generate a broad spectrum of bioactive chemicals and are the most significant source of mycoherbicides [176]. *C. eragrostidis*, for example, generates phytotoxic compounds, such dehydrocurvularin, helminthosporin, and curvularin, all used to control weeds [203]. About half of the metal was recovered from the uranium ore using the fungus *C. clavata* strain UC2F5 [204]. The *C. clavata* strain UC1FMGY was discovered to be acidophilic and capable of 50 percent extraction at a mine in Jaduguda, India [204]. *C. clavata* is hypothesized to have employed an indirect uranium-leaching method, combining uranium (UO22+), iron (Fe^2+^), oxygen (O_2_), and the hydronium ion (H3O^+^) in a mixture. Heavy metal cation absorption may occur in both dead and live cells since it is not reliant on metabolic processes. This demonstrates that the dead mycelia of fungus may be used for biosorption, making it a potential method for cleaning heavily loaded metal water [205]. For example, using oven dried fungal cell wall debris produced via lipid extraction from *Curvularia* sp. DFH1, the biosorption of Cd (II) and Zn (II) from artificial waste water at a concentration of 100 mg L^−1^ was examined, and after 1 h in solution, cell debris from *Curvularia* sp. strain DFH1 eliminated 85 percent of Cd (II) and 15 percent of Zn (II) [197]. Despite the lack of study in this area, using a fungal mycelium as a biosorbent might be a beneficial feature of environmentally friendly solutions for the cleanup of industrial waste.

## 14. Genomics of *Curvularia* and *Bipolaris* Species

Genomic research focuses on how genes function. The kingdom Fungi contains more than half of the currently known eukaryotic genome sequences. Additionally, 50% of the identified fungal genes are entirely new to science, proving that they are exclusive to fungi. In addition, the advantages of next-generation sequencing (NGS) technology, bioinformatics techniques, and the comparatively smaller quantities of fungal genomes compared to certain other eukaryotes have made the sequencing and evaluation of fungal genomics easier. There are gene families and orphan genes that have no known function. Understanding the functional role of these orphan genes is aided by high-throughput genomic and proteomic experiments [206,207]. The number of fully sequenced fungi has expanded significantly since the whole genome of the yeast *S. cereviseae* was sequenced and reported in 1996 [208], ushering in the age of fungal genomics. Fungal genomics has also significantly impacted the transcriptomes and proteomes of fungi at the genome level. Due to the availability of these enormous amounts of biological data, it is now possible to examine and categorize fungi systematically using their biological information. The US National Center for Biotechnology Information (NCBI) is a valuable source for scientists seeking genomics information and bioinformatics tools https://www.ncbi.nlm.nih.gov/, accessed on 13 October 2022.

The genetic fundamentals and genomics of *Cochliobolus*, *Bipolaris,* and *Curvularia* are largely unknown. However, there are 18 *Bipolaris* and 9 *Curvularia* species with whole-genome sequence data available in the NCBI database. These species’ genomes are variable in size, ranging from 31.2 Mb (*B. zeicola* Coccca1) to 43.9 Mb (*B. mydis* KET7) (Table 7). Similar variation was observed in the guanine–cytosine (GC) content, which varies from 48.1% (*B. sorokiniana Sacc*) to 54.2% (*B. mydis* KET7). Only four of these genomes, all from *B. sorokiniana*, have chromosomal-level assembly; the remaining genomes are only available just at scaffold and contig levels. Only nine of these genomes (eight *Bipolaris* and one *Curvularia*) were annotated, while the annotations for the remaining genomes were not available in the NCBI database. The number of protein-coding genes encoded by these annotated genomes ranged from 10,755 to 13,316 genes in *B. sorokiniana* Sacc and *B. maydis* C5, respectively (Table 7). Genomics analysis of three species, *C. lunata* [209], *B. papendorfii* UM_226 [210], and *Bipolaris cookei* [211], was reported. During the analysis, it was reported that *C. lunata* evolved from *B. maydis* (*C. heterostrophus*) and *C. lunata* and that *B. maydis* has a similar proportion of protein-encoding genes that are highly homologous to experimentally proven pathogenic genes from the pathogen–host interaction database [209]. The study of all these genomes aimed to identify the mechanisms behind pathogenesis in these species. At the genomic level, there is no information available for plant growth promotion and biotic or abiotic stress tolerance, etc. The growing number of whole genome sequences makes it much easier than ever to study the biology and evolution of *Curvularia* and *Bipolaris* species. The availability of genome sequences will help catalyze the development of genome-wide functional studies for many of these important fungal species.

## 15. Conclusions

In this review, we peruse the diversity, cultivation, lifestyle, and unique beneficial abilities of *Curvularia* and *Bipolaris* species in agriculture, environmental biotechnology, bioremediation, industrial enzymes, and mycoherbicide production. *Curvularia* and *Bipolaris* also perform various functions, including eliminating environmental contaminants, producing highly beneficial phytohormones, and continuing to survive as epiphytes, endophytes, and saprophytes. *Bipolaris* and *Curvularia*, as well as others, can be employed in the economy for knowledge-based exploitation, but this requires coordinated research and innovation efforts to develop programs that will enrich research institutions. The utilization of *Bipolaris* and *Curvularia* in modern research requires genome sequencing and analyses of key signature genes between species and strains. The results of genome analysis can help advance synthetic biology techniques, such as CRISPR/Cas9, which allow precise genome editing in improving the function of target genes. For the best possible application in the economy, *Bipolaris* and *Curvularia* research must also be focused on learning more about the physiology governing extracellular secretions, morphological transformations that take place in bioreactors, genes responsible for plant growth promotion, pathogenies, and mycoherbicides.

## Figures and Tables

**Table 1:** *Cochliobolus*, *Curvularia,* and *Bipolaris* species as endophytes.

Species	Accession Number	Host	Plant Part	References
*Cochliobolus intermedius*		Banana (*Musa* spp.)	Leaves	[63]
*Bipolaris papendorfii*		Banana (*Musa* spp.)	Leaves	[64]
*Bipolaris sp.*		Banana (*Musa* spp.)	Leaves	[65]
*Bipolaris cyanodontis*		*Solanum lycopersicum*	leaves	[66]
*Bipolaris cyanodontis*		*Musa* sp.	leaves	[66]
*Bipolaris sorokiniana*		*Triticum aestivum*	leaves, glumes	[55,66]
*Bipolaris* sp.		*Triticum aestivum*	Leaves, stem	[55]
*Cochliobolus australiensis*		*Musa* sp.	Leaves	[67,68]
*Cochliobolus sativus*		*Triticum aestivum*	Leaves	[69]
*Cochliobolus* sp. (UFMGCB-555)	EU684269	*Piptadenia adiantoides*	Leaves	[70]
*Cochliobolus* sp.(G2-20)	GQ461566.1	*Hevea brasiliensis*	Leaves	[71]
*Curvularia affinis*	KJ909780	*Elaeodendron glaucum*	Leaves	[53]
*Curvularia brachyspora*		*Thalassia testudinum*	Leaves	[72]
*Curvularia clavata*		*Adhatoda zeylanica*	Leaves	[73]
*Curvularia clavata*		*Bauhinia phoenicea*	Bark, leaves	[73]
*Curvularia clavata*		*Callicarpa tomentos*	Bark, leaves	[74]
*Curvularia geniculata*		*Bauhinia phoenicea*	Bark, leaves	[73]
*Curvularia inaequalis*		*Holcus lanatus*	Leaves	[75,76]
*Curvularia lunatus*		*Callicarpa tomentosa*	Bark, leaves	[73]
*Curvularia lunatus*		*Bauhinia phoenicea*	Bark, leaves	[73]
*Curvularia lunatus*		*Callicarpa tomentosa*	Leaves	[73]
*Curvularia lunatus*		*Ficus benghalensis*	Leaves	[53]
*Curvularia lunatus*		*Azadirachta indica*	Leaves	[77]
*Curvularia lunatus*		*Thalassia testudinum*	Leaves	[72]
*Curvularia lunatus*		*Triticum aestivum*	Leaves, glumes, grains	[55]
*Curvularia pallescens*		*Callicarpa tomentosa*	Stem, leaves	[73]
*Cochliobolus spicifer*		*Triticum aesitivum*	Grains	[55]
*Curvularia oryzae*		*Erythroxylon monogynum*	Leaves	[53]
*Curvularia tuberculata (516)*	(AC) 10336	*Elaeodendron glaucum*, *Dalbergia latifolia*	Leaves	[78]
*C. asiatica*	JX256424	*Panicum* sp.	Leaves	[79]
*C. beerburrumensis*	MH414894	*Eragrostis bahiensis*	Leaves	[79]
*C. cactivora*	MN688803	Member of Cactaceae	Leaves	[79]
*C. chiangmaiensis*	MF490814	*Zea mays*	Leaves	[79]
*C. coicis*	JN192373	*Coix lacryma*	Leaves	[79]
*C. cymbopogonis*	HG778985	*Yucca* sp.	Leaves	[79]
*C. dactyloctenii*	KJ415545	*Dactyloctenium radulans*	Leaves	[79]
*C. eragrosticola*	MH414899	*Eragrostis pilosa*	Leaves	[79]
*C. gladioli*	HG778987	*Gladiolus* sp.	Leaves	[79]
*C. heteropogonis*	JN192379	*Heteropogon contortus*	Leaves	[79]
*C. intermedia*	HG778991	*Avena versicolor*	Leaves	[79]
*C. ischaemi*	JX256428	*Ischaemum indicum*	Leaves	[79]
*C. microspore*	MF139088	*Hippeastrum striatum*	Leaf	[79]
*C. mosaddeghii*	MG846737	*Syzygium cumini*	Leaf	[79]
*C. neergaardii*	KJ415550	*Oryza sativa*	Leaves	[79]
*C. nodosa*	MF490816	*Digitaria ciliaris*	Leaves	[79]
*C. nodulosa*	JN192383	*Eleusine indica*	Leaves	[79]
*C. oryzae*	KP400650	*Oryza sativa*	Leaves	[79]
*C. ovariicola*	MN688809	*Eragrostis interrupta*	Leaves	[79]
*C. palmicola*	MF621582	*Acoelorrhaphe wrightii*	Leaves	[79]
*C. papendorfii*	KJ909774	*Acacia karroo*	Leaves	[79]
*C. paterae*	MN688810	*Triticum durum*	Seed	[79]
*C. perotidis*	JN192385	*Perotis rara*	Leaves	[79]
*C. petersonii*	MH414905	*Dactyloctenium aegyptium*	Leaves	[79]
*C. portulacae*	KJ415553	*Portulaca oleracea*	Leaves	[79]
*C. sorghina*	KJ415558	*Sorghum bicolor*	Leaves	[79]
*C. sporobolicola*	MH414908	*Sporobolus australasicus*	Leaves	[79]
*C. thailandica*	MH275057	*Pandanus* sp.	Leaves	[79]
*C. trifolii*	HG779023	*Trifolium repens*	Leaves	[79]
*C. tuberculate*	JX256433	*Zea mays*	Leaves	[79]
*C. xishuangbannaensis*	MH275058	*Pandanus* sp.	Leaves	[79]
*Bipolaris maydis*	KJ909769	*Zea mays*	Leaves	[79]
*B. sorokiniana*	KJ922381	*Hordeum* sp.	Leaves	[79]
*Curvularia affinis*	HG778981.1	*G. ulmifolia*	Leaves	[79]
*Curvularia lunata*	OP265394	*Solanaum nigram*	Root	[80]

**Table 2:** *Cochliobolus* and its anamorph as saprobes.

Species	Host	Plant Part	References
*Cochliobolus* sasae	*Sasa tambaensis* (Poaceae)	Dead leaves	[85]
*Bipolaris ellisii(ZG-9)*	*Pinus khasya*	Dead leaves	[86]
*Bipolaris maydis*	*Zea mays*	Seeds	[87]
*Curvularia cymbopogonis*	*Pinus caribaea*	Damping-off of seedlings	[88]
*Curvularia eragrostidis*	*Pinus caribaea*	Root rot	[89]
*Curvularia eragrostidis*	*Pinus khasya*	Decaying and fallen needles	[86]
*Curvularia eragrostidis*	*Phaseolus vulgaris*	Pod rot	[90]
*Curvularia eragrostidis*	*Pandanus*	Decaying leaves	[54]
*Curvularia eragrostidis*	*Pandanus odoratissimus*	Decaying leaves	[54]
*Cochliobolus geniculatus*	*Phaseolus vulgaris*	Rotting pods	[90]
*Curvularia intermedia*	*Zea mays* subsp. *Mays*	Dead leaves	[91]
*Curvularia intermedia*	*Zea mays*	Dead leaves	[92]
*Curvularia lunta*	*Bambusa* sp.	Dead clumps	[83]
*Curvularia pallescens*	*Phaseolus vulgaris*	Pod rot	[90]
*Curvularia pallescens*	*Pinus taiwanensis*	Root rot	[89]
*Curvularia tuberculatus*	*Pinus caribaea*	From damped-off seedlings	[88]
*Curvularia verruculosus*	*Pinus caribaea*	From damped-off seedlings	[88]
*Curvularia* sp.	*Magnolia liliifera*	Woody litter	[82]
Cochliobolus sitharamii	*Bambusa* sp. (Poaceae)	Leaves	[45]

**Table 3:** Fungal species with plant growth-promoting characteristics.

Species	Accession Number	References
*Cochliobolus lunatus*	MK611790	[99]
*Bipolaris* sp.		[100]
*C. geniculata*	KX656363	[100,101]
*Curvularia pallescens*	KJ534376	[93]
*Cochliobolus lunatus*	KJ534378	[93]
*Cochliobolus setariae* IFO 6635		[102]
*Curvularia lunata* AR11	MZ145250	[56]
*Bipolaris* sp. CSL-1		[103]
*Bipolaris* spp.		[104]

**Table 4:** Some secondary metabolites isolated from *Curvularia* and *Bipolaris* and their classes and biological activities.

Chemical Classes	Natural Products	Biological Activities	Fungi	References
Alkaloids	curvulamine	antimicrobial, anti-inflammatory	*Curvularia* sp. IFBZ10	[126]
	curindolizine	anti-inflammatory	*Curvularia* sp. IFBZ10	[126]
	cytochalasin B		*Curvularia lunata*	[127]
	curvupallide A		*Curvularia pallescens*	[128]
	curvupallide B			
	curvupallide C			
	spirostaphylotrichin A			
	spirostaphylotrichin C			
	spirostaphylotrichin D			
	spirostaphylotrichin Q			
	spirostaphylotrichin R			
	spirostaphylotrichin U			
	spirostaphylotrichin V			
	bipolaramide		*Bipolaris sorokiniana*	[129]
Peptides	victorin C	phytotoxicity	*Bipolaris victoriae*	[100]
	HC-toxin		*Bipolaris arbonum*	[100]
Polyketides	apralactone A	cytotoxicity	*Curvularia* sp.	[130]
	(þ)-(15R)-10,11-E-dehydrocurvularin			
	(þ)-(15R)-12-hydroxy-10,11-E-dehydrocurvularin			
	(þ)-(11R,15R)-11-hydroxycurvularin			
	(þ)-(15R)-12-oxocurvularin			
	curvularin	natural biopesticides, antimicrobial	*Cochliobolus* sp.	[71]
	curvularide A, C, D, E		*Curvularia geniculata*	
	curvularide B	antifungal property	*Curvularia geniculata*	[131]
	curvulide A			
	curvulide B1			
	curvulide B2			
	6-chlorodehydrocurvularin	leishmanicidal activity	*Cochliobolus spicifer*	[70]
	modiolide A			
	1,14-dihydroxy-6-methyl-6,7,8,9,10,10a,14,14a-octahydro-1H-benzo[f][1]oxacyclododecin-4(13H)-one.	antioxidant, anticancer	*Curvularia trifolii*. US/US/06	[132]
	5-methoxy-4,8,15-tri methyl-3,7-dioxo-1,3,7,8,9,10,11,12,13,14,15	anti-inflammatory, antioxidant		
	15a dodecahydrocyclododeca [de] isochromene -15-carboxylic acid			
	curvulinic acid		*Curvularia siddiqui*	[133]
	curvulol			
	methyl2-acetyl-3,5 dihydroxyphenylacetate		*Curvularia siddiqui*	
	methyl2-acetyl-5-hydroxy-3-methoxyphenylacetate		*Curvularia lunata*	
	curvulin			
	11-a-methoxycurvularin	antibacterial antifungal	*Curvularia oryzae*	[134]
	(S)-5-ethyl-8, 8-dimethylnonanal	antilarval		
	bipolarinone		*Bipolaris* sp. PSU-ES64	[135]
	bipolarilide			
	paecilin B			
	(5S, 10aR)-gonytolide C			
	T-toxin		*Bipolaris heterostrophus*	[136]
	cochliomycin A	antibacterial	*Bipolaris lunatus* (TA26-46)	[137]
	cochliomycin B			
	cochliomycin C			
	cochliomycin D	antifouling		
	cochliomycin E			
	cochliomycin F	antifouling		
	zeaenol			
	LL-Z1640-1	cytotoxicity, antifouling		
	LL-Z1640-2	fungicide		
	paecilomycin F			
	(70E)-60-oxozeaenol	antifouling		
	(70E)-60-oxozeaenol antifouling			
	aigialomycin B			
	deoxy-aigialomycin C	antifouling		
	cochliobolic acid		*Bipolaris lunatus*	[138]
	ethyl 2-[(3,5-dihydroxy)phenyl] acetate		*Bipolaris* sp. PSU-ES64	[135]
	(2-carboxy-3-hydroxy-5-methoxyphenyl) acetic acid			
	4-epiradicinol	antibacterial		
	radicinol	phytotoxicity	*Bipolaris lunata*	[139]
	epi-radicinol	phytotoxicity		
	radicinin	phytotoxicity	*Bipolaris* sp.	[140]
	lunatoic acid A	antifungal	*Bipolaris lunata*	[141]
	lunatoic acid B			
	a-acetylorcinol			
Quinones	cynodontin		*Curvularia lunata*	[142]
	lunatin		*Curvularia lunata*	[143]
	cytoskyrin A			
	mitorubrinic acid A		*Bipolaris lunatus*	[144]
	mitorubrinic acid B			
	chrysophanol		*Bipolaris* sp. PSU-ES64	[135]
	emodin			
Terpenes	abscisic acid		*Curvularia lunata*	[143]
	zaragozic acid A		*Curvularia lunata*	[145]
	3a-hydroxy-5b-chol- 11-en-24-oic acid		*Curvularia* sp.	[146]
	cochlioquinone A	leishmanicidal	*Bipolaris* sp.	[70]
	isocochlioquinone A	leishmanicidal		
	cochlioquinone B		*Bipolaris miyabeanus*	[124]
	isocochlioquinone C		*Bipolaris oryzae*	[147]
	anhydrocochlioquinone A			
	ophiobolin A	cytotoxicity, antimalarial	*Bipolaris heterostrophus* race O	[148]
	3-anhydroophiobolin A		*Bipolaris oryzae*	[147]
	6-epi-ophiobolin A			
	6-epi-3-anhydroophiobolin A			
	ophiobolin B			
	-epi-3-anhydroophiobolin B			
	ophiobolin I			
	3-anhydro-6-hydroxy-ophiobolin A	antiproliferative	*Bipolaris oryzae*	[149]
	cis-sativenediol		*Bipolaris setariae*	[102]
	isosativendiol			
	IFO 6635 aversion factor	antifungal	*Bipolaris setariae*	[150]
	isosativenetriol	anti-inflammatory, anti-diabetic	*Cochliobolus* sp.	[71]
	anhydrocochlioquinone A	anti-tumor compound	*Bipolaris oryzae*	[147,151]
	cochlioquinone A1	anti-angiogenic agent	*Bipolaris zeicola* (FIP 532)	[151,152],
	cochlioquinone A1	anti-angiogenic agent	*B. zeicola* (AR5166)	[151,152]
	cochlioquinone A1	anti-angiogenic agent	*B. zeicola* (AR 5168)	[151,152]
	cochlioquinones A, B		*Cochliobolus miyabeanus*	[124]
	spiciferone A	antiviral property	*Cochliobolus spicifer*	[153]
	cochlioquinone A, isocochlioquinone A	leishmanicidal activity	*Cochliobolus* sp.	[70]

**Table 5:** Biological activities of *Curvularia* and *Bipolaris* species.

Species	Sources	Biological Activities	References
*Curvularia lunata*	*Cymbopogon caesius*	Antibacterial: *Staphylococcus aureus*. Antifungal: *Candida albicans*	[156]
*Curvularia* sp.	*Catharanthus roseus*	Antioxidant	[22]
*Curvularia* sp.	*Phyllostachys edulis*	Antibacterial: *Bacillus subtilis*, *Listeria* monocytogenes, *Salmonella* bacteria. Antifungal: *Candida albicans*.	[157]
*Curvularia* sp.	*Moso bamboo* (seeds)	Antibacterial: *Listeria monocytogenes*, *Salmonella* bacteria. Antifungal: *Candida albicans*.	[157]
*Curvularia* sp. (D12)	*Garcinia* spp.	Antibacterial: *Mycobacterium tuberculosis*	[158]
*Curvularia lunata*	*Litchi chinensis*	Phytotoxicity	[100,127]
*Curvularia tuberculata*	*Marine algea*	Antibacterial: *Staphylococcus aureus*, *Escherichia coli*, *Pseudomonas aeruginosa*. Antioxidant	[159]
*Curvularia pallescens*	*Laguncularia racemosa*	Antibacterial: *Staphylococcus aureus*, *Bacillus subtilis*, *Micrococcus luteus*, *Escherichia coli*	[160]
*Curvularia* spp.	*Ipomoea carnea*	Leishmanicidal	[70]
*Bipolaris geniculatus*, *B. spicifer*	*Cynodon dactylon*	Antibacterial: *Enterococcus faecalis*	[161]
*B. lunatus*	*Dactyloctenium aegyptium*	Antibacterial: *Salmonella enterica*, *Staphylococcus aureus*. Antifungal: *Candida albicans*. Antioxidant	[161]
*B. hawaiiensis*		Antibacterial: *Salmonella enterica*, *Staphylococcus aureus*. Antifungal: *Candida albicans*. Antioxidant	[161]
*Bipolaris*	*Costus spiralis*	Antibacterial: *Pseudomonas aeruginosa*, *Salmonella enterica* subsp. enterica serovar *Typhi*, *Bacillus subtilis*, and *Enterococcus faecalis*. Antifungal: *Candida albicans*, *C. parapsilosis*. Antioxidant	[162]
*Bipolaris*	*Plumeria obtusifolia*	Antibacterial: *Pseudomonas aeruginosa*.	[163]

**Table 6:** *Cochliobolus*, *Bipolaris*, and *Curvularia* species function as biological control agents.

Species	Accession Number	Host/Plant	References
*Cochliobolus* sp.	DQ836798	*Grapevine*	[140]
*Bipolaris ravenelii*		*Sporobolus* spp.	[182]
*Bipolaris setariae*		*Eleusine indica*	[183]
*Bipolaris* sp. (*B. zeicola*)	GU046562	*Microstegium vimineum*	[184]
*Curvularia eragrostidis*		*Digitaria sanguinalis*	[185]
*Curvularia lunata*		*Alisma plantago-aquatica*	[186]
*Curvularia lunata*		*Echinochola* spp.	[187]
*Curvularia tuberculata*		*Cyperus difformis*	[188]
		*Cyperus iridis*	[188]
		*Fimbristylis miliacea*	[188]
*Cochliobolus australiensis*	JX960591	*Pennisetum ciliare*, or *Cenchrus ciliaris*	[27,100]

**Table 7:** Whole genome sequences of *Bipolaris* and *Curvularia* species available in the NCBI database.

Name	Strain	Assembly	Level	Size (Mb)	GC%	Scaffolds	CDS	Genes	rRNA	tRNA
*Bipolaris cookei*	LSLP18.3	GCA_002286855.1	Scaffold	36.1	49.7	320	0	0	0	0
*Bipolaris sorokiniana*	BS112	GCA_004329375.1	Contig	37.3	49.4	43	0	0	0	0
*Bipolaris sorokiniana*	Shoemaker	GCA_013416765.1	Scaffold	34.3	48.1	96	10,755	10,755	0	0
*Bipolaris sorokiniana*	ND90Pr	GCA_000338995.1	Scaffold	34.4	49.8	154	12,210	12,304	0	94
*Bipolaris oryzae*	TG12bL2	GCA_001675385.1	Scaffold	31.6	50.5	1640	0	0	0	0
*Bipolaris oryzae*	ATCC 44560	GCA_000523455.1	Scaffold	31.3	50.5	619	12,002	12,002	0	0
*Bipolaris victoriae*	FI3	GCA_000527765.1	Scaffold	32.8	50.1	676	12,882	12,882	0	0
*Bipolaris zeicola*	GZL10	GCA_016906865.1	Contig	36.1	50.6	23	0	0	0	0
*Bipolaris zeicola*	Cocca1	GCA_023079205.1	Scaffold	31.2	50.8	828	0	0	0	0
*Bipolaris maydis*	ATCC 48331	GCA_000354255.1	Scaffold	32.9	50.7	207	12,705	12,809	0	104
*Bipolaris zeicola*	26-R-13	GCA_000523435.1	Scaffold	31.2	50.8	844	12,853	12,853	0	0
*Bipolaris maydis*	BM1	GCA_019454015.1	Contig	36.2	49.1	27	11,026	11,162	26	110
*Bipolaris maydis*	KET7	GCA_023087585.1	Scaffold	43.9	54.2	278	0	0	0	0
*Bipolaris maydis*	C5	GCA_000338975.1	Scaffold	36.4	49.8	68	13,316	13,456	0	140
*Bipolaris sorokiniana*	WAI2411	GCA_008452715.1	Chromosome	36.2	49.4	21	0	0	0	0
*Bipolaris sorokiniana*	WAI2406	GCA_008452705.1	Chromosome	36.8	49.4	21	0	0	0	0
*Bipolaris sorokiniana*	BRIP10943a	GCA_008452735.1	Chromosome	36.9	49.5	22	0	0	0	0
*Bipolaris sorokiniana*	BRIP27492a	GCA_008452725.1	Chromosome	35.2	49.4	19	0	0	0	0
*Curvularia* sp.	IFB-Z10	GCA_002161795.1	Scaffold	33.0	50.4	136	0	0	0	0
*Curvularia* sp.	ZM96	GCA_022457065.1	Contig	35.5	50.7	54	0	0	0	0
*Curvularia geniculata*	P1	GCA_016162275.1	Contig	32.9	50.7	753	0	0	0	0
*Curvularia geniculata*	W3	GCA_002982235.1	Scaffold	33.5	50.6	737	0	0	0	0
*Curvularia papendorfii*	UM 226	GCA_000817285.1	Contig	33.3	50.6	374	0	0	0	0
*Curvularia lunata*	W3	GCA_005212705.1	Scaffold	33.5	50.6	737	0	0	0	0
*Curvularia kusanoi*	30M1	GCA_011058905.1	Scaffold	33.3	52.1	107	11,490	11,686	0	196
*Curvularia eragrostidis*	C52	GCA_020744315.1	Contig	36.8	52.1	3600	0	0	0	0
*Curvularia lunata*	CX-3	GCA_000743335.1	Scaffold	35.4	50.1	340	0	0	0	0

## Data Availability

Not applicable.

## Supplementary Materials

The following supporting information can be downloaded at: https://www.mdpi.com/article/10.3390/jof9020254/s1, Table S1: List of *Curvularia* species available in Mycobank database; Table S2: Tree-aligment; Table S3: List of *Chochliobolus*, *Bipolaris* and *Curvularia* speceis available in Westerdijk institute of the Fungal Biodiversity Culture center; Table S4: *Cochliobolus*, *Curvularia* and *Bipolaris* sp. in stress tolerance; Table S5: *Cochliobolus* species in industrial biotransformation.

## Abbreviations

IAA	Indole acetic acid
GA	Gibberellic acid
PCB	Polychlorinated biphenyls
PAH	Polyaromatic hydrocarbons
rDNA	Ribosomal deoxyribonucleic acid
ITS	Internal transcribed spacer
GAPDH	Glyceraldehyde 3-phosphate dehydrogenase
NCBI	National Center for Biotechnology Information
ML	Maximum likelihood
sp	Species
P	Phosphorus
MIC	Minimum inhibitory concentration
RNA	Ribonucleic acid
cThTv	Curvularia thermal tolerance virus
POME	Palm oil mill effluent
LME	Lignin-modifying enzyme
ROS	Reactive oxygen species
USD	United States Dollar
Mg	Milligram
L	Liter
NGS	Next-generation sequencing
GC	Guanine–cytosine
CRISPR	Clustered regularly interspaced short palindromic repeats
Cas9	CRISPR-associated protein 9
