# Supplementary material for: Diversity, Lifestyle, Genomics, and Their Functional Role of Cochliobolus, Bipolaris, and Curvularia Species in Environmental Remediation and Plant Growth Promotion under Biotic and Abiotic Stressors
Source: J Fungi (Basel). 2023 Feb 14;9(2):254. doi: 10.3390/jof9020254 (PMC9962790; doi:10.3390/jof9020254)
Supplement: Supplementary file 1 [file jof-09-00254-s001.zip › jof-2061993-supplementary/Table S4.pdf]

Table S4. ***Cochliobolus, Curvularia and Bipolaris*** **sp. in stress tolerance**

species	Stress	references
Cochliobolus lunatus	salinity stress	(Bibi et al., 2019)
Curvularia cryptic	Thermal stress	(Bengyella et al., 2019)
Curvularia protuberata	Thermal stress	(Redman, Sheehan, Stout, Rodriguez, & Henson, 2002)
Curvularia crepinii	Thermal stress	(Zhou et al., 2015)
Curvularia protuberrata	Thermal stress	(Zhou et al., 2015)
Curvularia species	Drought stress	(Adhikari et al., 2022; Sangamesh et al., 2018)
C. clavata	Low ph stress/uranium extraction efficiency	(Bengyella et al., 2019)
C. lunatus	crude oil stress/ breakdown of polycyclic aromatic hydrocarbon	(Al-Nasrawi, 2012; Bengyella et al., 2019)

Adhikari, A., Khan, M., Imran, M., Lee, K., Kang, S., Shin, J., . . . Lee, I. (2022). The Combined Inoculation of Curvularia lunata AR11 and Biochar Stimulates Synthetic Silicon and Potassium Phosphate Use Efficiency, and Mitigates Salt and Drought Stresses in Rice. *Frontiers in plant science, 13*.

Al-Nasrawi, H. (2012). Biodegradation of crude oil by fungi isolated from Gulf of Mexico. *J Bioremed Biodegrad, 3*(4), 147-152.

Bengyella, L., Iftikhar, S., Nawaz, K., Fonmboh, D. J., Yekwa, E. L., Jones, R. C., . . . Roy, P. (2019). Biotechnological application of endophytic filamentous bipolaris and curvularia: a review on bioeconomy impact. *World Journal of Microbiology and Biotechnology, 35*(5), 1-14.

Bibi, N., Jan, G., Jan, F. G., Hamayun, M., Iqbal, A., Hussain, A., . . . Khushdil, F. (2019). Cochliobolus sp. acts as a biochemical modulator to alleviate salinity stress in okra plants. *Plant physiology and biochemistry, 139*, 459-469.

Redman, R. S., Sheehan, K. B., Stout, R. G., Rodriguez, R. J., & Henson, J. M. (2002). Thermotolerance generated by plant/fungal symbiosis. *Science, 298*(5598), 1581-1581.

Sangamesh, M., Jambagi, S., Vasanthakumari, M., Shetty, N. J., Kolte, H., Ravikanth, G., . . . Uma Shaanker, R. (2018). Thermotolerance of fungal endophytes isolated from plants adapted to the Thar Desert, India. *Symbiosis, 75*(2), 135-147.

Zhou, W.-N., White, J. F., Soares, M. A., Torres, M. S., Zhou, Z.-P., & Li, H.-Y. (2015). Diversity of fungi associated with plants growing in geothermal ecosystems and evaluation of their capacities to enhance thermotolerance of host plants. *Journal of Plant Interactions, 10*(1), 305-314.
