# Supplementary material for: Diversity, Lifestyle, Genomics, and Their Functional Role of Cochliobolus, Bipolaris, and Curvularia Species in Environmental Remediation and Plant Growth Promotion under Biotic and Abiotic Stressors
Source: J Fungi (Basel). 2023 Feb 14;9(2):254. doi: 10.3390/jof9020254 (PMC9962790; doi:10.3390/jof9020254)
Supplement: Supplementary file 1 [file jof-09-00254-s001.zip › jof-2061993-supplementary/Table S5.pdf]

**Table S5. *Cochliobolus* species in industrial biotransformation**

| Fungal species | Host | Biotehnological application | References |
|---|---|---|---|
| *Curvularia sp. strain DHE5* | Soil | Production of extracellular lipase | (El-Ghonemy, El-Gamal, Tantawy, & Ali, 2017) |
| *Curvularia kusanoi strain L7* | Natural substrates | *Produced laccase with lignocel lulolytic potential of 2800 U L-1 enzymatic activity, 544.74 U g-1 specific activity and stable at 40 °C* | (Valiño Cabrera, Alberto Vázquez, Dustet Mendoza, & Albelo Dorta, 2020) |
| *Cochliobolus lunatus* | Unknown | Biotransformation of the anti diabetic agent corosolic acid (2α,3β-dihydroxyurs-12-en-28-oic acid) use for treatment of to treat diabetes, polyuria andpolydipsia | (Feng, Li, Zhang, Chu, & Luan, 2014) |
| *Curvularia spp.* | Ipomoea carnea | *Antibiotics production against Bacillus subtilis and Escherichia coli* | (Tayung, Sarkar, & Baruah, 2012) |
| *Curvularia pallescens* | Laguncularia racemosa | Antibiotics production against Staphylococcus aureus, Bacillus subtilis, Micrococcus luteus and Escherichia coli | (Silva, Almeida, Arruda, & Gusmao, 2011)Silva et al. (2011) |
| *Curvularia sp.* | Garcinia spp. | Antibiotics production against Mycobacterium tuberculosis | (Phongpaichit et al., 2007) |
| *Cochliobolus australiensis* | Pennisetum ciliare or Cenchrus ciliaris | Bioherbicides potential observed with cochliotoxin | (Masi et al., 2017)Masi et al. (2017a, b) |
| *Cochliobolus lunatus* | Mycobacterium smegmatis | Biotransformation of Mycobacterium smegmatis with Cochliobolus lunatus gene17β-hydroxysteroid: NADP 17-oxidoreductase for the production of testosterone | (Fernández - Cabezón, Galan, & García, 2017) |

El-Ghonemy, D. H., El-Gamal, M. S., Tantawy, A. E., & Ali, T. H. (2017). Extracellular alkaline lipase from a novel fungus Curvularia sp. DHE 5: Optimisation of physicochemical parameters, partial purification and characterisation. *Food Technology and Biotechnology, 55*(2), 206.

Feng, X., Li, D.-P., Zhang, Z.-S., Chu, Z.-Y., & Luan, J. (2014). Microbial transformation of the anti-diabetic agent corosolic acid. *Natural Product Research, 28*(21), 1879-1886.

Fernández ‐ Cabezón, L., Galan, B., & García, J. L. (2017). Engineering Mycobacterium smegmatis for testosterone production. *Microbial Biotechnology, 10*(1), 151-161.

Masi, M., Meyer, S., Clement, S., Pescitelli, G., Cimmino, A., Cristofaro, M., & Evidente, A. (2017). Chloromonilinic acids C and D, phytotoxic tetrasubstituted 3-chromanonacrylic acids isolated from Cochliobolus australiensis with potential herbicidal activity against buffelgrass (Cenchrus ciliaris). *Journal of natural products, 80*(10), 2771-2777.

Phongpaichit, S., Nikom, J., Rungjindamai, N., Sakayaroj, J., Hutadilok-Towatana, N., Rukachaisirikul, V., & Kirtikara, K. (2007). Biological activities of extracts from endophytic fungi isolated from Garcinia plants. *FEMS Immunology & Medical Microbiology, 51*(3), 517-525.

Silva, M., Almeida, A., Arruda, F., & Gusmao, N. (2011). Endophytic fungi from brazilian mangrove plant Laguncularia racemosa (L.) Gaertn.(Combretaceae): their antimicrobial potential. *Science against microbial pathogens: communicating current research and technological advances, 2*, 1260-1266.

Tayung, K., Sarkar, M., & Baruah, P. (2012). Endophytic fungi occurring in Ipomoea carnea tissues and their antimicrobial potentials. *Brazilian Archives of Biology and Technology, 55*, 653-660.

Valiño Cabrera, E. C., Alberto Vázquez, M., Dustet Mendoza, J., & Albelo Dorta, N. (2020). Producción de enzimas lignocelulasas de Trichoderma viride M5-2 en salvado de trigo (Triticum aestivum) y purificación de sus lacasas. *Cuban Journal of Agricultural Science, 54*(1), 55-66.
